# Feasibility and Safety of the Craniocaudal Approach for Superior Sulcus Lesions of the Thorax

**DOI:** 10.1007/s00270-021-02844-y

**Published:** 2021-05-11

**Authors:** Yuji Koretsune, Miyuki Sone, Yasuaki Arai, Shunsuke Sugawara, Chihiro Itou, Shintaro Kimura, Masahiko Kusumoto

**Affiliations:** Department of Diagnostic Radiology, National Cancer Center Hospital, 5-1-1, Tsukiji, Chuo-ku, Tokyo 1040045 Japan

**Keywords:** Craniocaudal approach, Drainage, Biopsy

## Abstract

**Purpose:**

To evaluate the feasibility and safety of the craniocaudal approach for superior sulcus lesions of the thorax.

**Material and Methods:**

Between October 2010 and December 2020, the data from 22 consecutive patients who underwent drainage or biopsy using a craniocaudal trajectory were retrospectively reviewed. The craniocaudal approach was applied for patients in which the fluid collection or tumor was limited to the superior thoracic sulcus lesion or otherwise inaccessible owing to intervening structures such as pleural dissemination. The indications for this procedure were drainage in 20 patients and biopsy in 2 patients. Technical success, procedure time, complications, and clinical success were evaluated.

**Results:**

Technical and clinical success were achieved in all patients, and no major complications were found. The median procedure time was 25 min (range 15–40 min). This procedure was performed with fluoroscopic guidance in 11 patients and ultrasound guidance in 11 patients. The routes of needle passage were the first intercostal space (*n* = 16), the second intercostal space (*n* = 5), and between the clavicle and the first rib (*n* = 1).

**Conclusion:**

The craniocaudal approach for superior sulcus lesions might be a safe and feasible option for patients in which the conventional intercostal approach is difficult.

**Level of Evidence:**

Retrospective cohort study. Level 4.

## Introduction

Pleural effusion and empyema are common conditions that may require thoracic drainage or pleurodesis [1]. An intercostal approach is most widely applied for percutaneous thoracic drainage with the trocar or Seldinger’s technique [2, 3]; however, thoracic superior sulcus lesions are sometimes difficult to access using the conventional intercostal approach because of the intervening structures, such as the subclavian artery and vein, scapula, and clavicle. Takizawa et al. [4] reported two cases of drainage in which the needle punctured the skin from just above the posterior third rib to the apical zone. That is called the axial puncture approach. Although this approach may be beneficial, the feasibility, safety, and efficacy have not been fully evaluated. Thus, this study aimed to evaluate the safety and feasibility of the craniocaudal approach.

## Material and Methods

### Patients

This single-institution, retrospective, and observational study was performed in accordance with the Declaration of Helsinki and approved by the Institutional Ethics Committee. Informed consent for participation in this study was waived due to the retrospective nature of the study. Written informed consent for the procedure was obtained from all patients.

Between October 2010 and December 2020, 22 consecutive patients (15 males and 7 females, median age 65.5 years, range 36–86 years) who underwent drainage or biopsy using the craniocaudal approach were included. The most common reason for employing the craniocaudal approach was difficulty with conventional intercostal access (*n* = 17). Difficulty with conventional intercostal approach was defined by the operator’s subjective judgment. The demographic and clinical characteristics of the patients are summarized in Table 1.

**Table1:** Demographic and clinical characteristics of the 22 patients

Characteristics	No. of patients (*N* = 22)
Age, *y*	
Median (range)	65.5 (36–86)
Sex	
Male	15 (68.2%)
Female	7 (31.8%)
Diagnosis	
Lung cancer	7 (31.8%)
Breast cancer	3 (13.6%)
Colon cancer	2 (9.1%)
Angiosarcoma	2 (9.1%)
Esophagus cancer	2 (9.1%)
Osteosarcoma	1 (4.5%)
Bladder cancer	1 (4.5%)
Prostate cancer	1 (4.5%)
Malignant melanoma	1 (4.5%)
Malignant fibrous histiocytoma	1 (4.5%)
Malignant glioma	1 (4.5%)
Purpose of procedures	
Drainage	
Pleural effusion	10 (45.5%)
Empyema	10 (45.5%)
Biopsy	2 (9.1%)
Indication of axial puncture	
Target confined to the superior sulcus lesion	18 (78.3%)
Other routes not feasible because of the tumor	5 (21.7%)

### Procedures

**Fig. 1:**
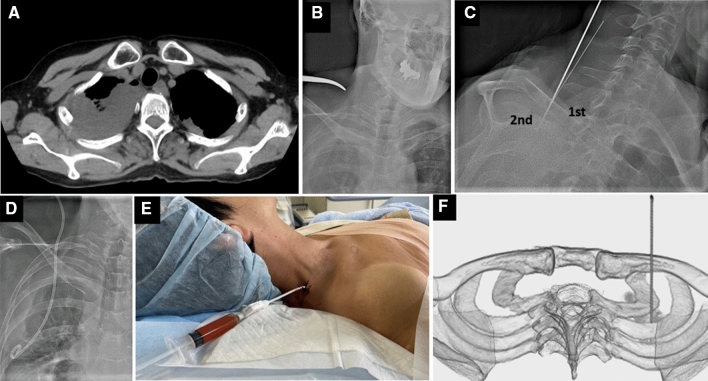
**A** A female patient in her sixties with right pleural effusion after a lobectomy for lung cancer. **B** A surgical Pean forceps pointing at the puncture site located just above the posterior first rib. **C, D, E** A 17-gauge needle is passed through first intercostal space with 60 ° right anterior oblique projection and an 8.5 Fr catheter (Dawson-Mueller Multipurpose Drainage Catheter; Cook, Bloomington, IN, USA) was inserted. **F** A 3D-CT showing the needle trajectory

All procedures were performed by 10 interventional radiologists with 3–24 years of experience in interventional radiology in the angiography suite with a hybrid angio-computed tomography (CT) system. Each procedure was performed under local anesthesia. In most patients (*n* = 16/22), hydroxyzine (Atarax-P; Pfizer, NY, USA) and pentazocine (Pentazine; Daiichi Sankyo, Tokyo, Japan) were administered as procedural sedation and analgesia. The patient was placed in the supine or 15°–45° semi-erect position in the case of dyspnea. For fluoroscopic puncture, the method described in Takizawa’s article was primarily used [4]. A 17-gauge metallic Huber-point needle (PTC needle; Hakko, Chikuma, Japan) was inserted from the base of the neck to the superior sulcus lesion with fluoroscopic or ultrasound (US) guidance (TUS-300/Aplio300; Toshiba Medical Systems). The choice of modality was decided based on operator’s preference. Before and during the puncture, CT scan was performed and utilized as a guidance for the puncture direction. The superior sulcus lesion was accessed mainly through the first or second intercostal spaces because of the low risk of injury to vessels or nerves. Lateral or oblique views under fluoroscopy were effective in inserting the needle through the intercostal space. In the drainage cases, a 0.035-inch J-shaped guide wire (Fixed Core Wire Guide; Cook, Bloomington, IN, USA) was advanced into the cavity after the puncture of the needle, followed by the placement of a drainage catheter (Fig. 1). Prior to the removal of drainage catheters, the shrinkage of the cavities was confirmed via chest CT or radiograph.

### Study Outcomes

The primary outcome parameters was technical success, defined as the successful placement of the drainage catheter or successful collection of tissue samples with biopsy devices. Procedure time, complications, and clinical success were also evaluated. Clinical success was defined as the shrinkage of the drained cavity, followed by the removal of the catheter, or when the pathologists could make a diagnosis from the biopsy specimen. Complications were evaluated using the Cardiovascular and Interventional Radiological Society of Europe (CIRSE) classification system [5].

## Results

### Technical and Clinical Success

Both the technical and clinical success rates were 100% (*n* = 22/22). The craniocaudal approach was performed with fluoroscopic guidance in 11 patients and US guidance in 11 patients. The most common needle trajectory was through the first intercostal space (*n* = 16/22). In the 20 drainage cases, 20 fluid collections have resolved in total.

### Complications

Minor complications (CIRSE classification Grade 2) occurred in one case. In this patient, the normal lung parenchyma was mistakenly traversed. Transient hemoptysis occurred, but it disappeared in 24 h. The clinical outcomes and follow-up data are summarized in Table 2.

**Table 2:** Clinical outcomes and follow-up data

Parameters	No. of patients
	(*N* = 22)
Modality used	
Fluoroscopy	11 (50.0%)
Ultrasound	11 (50.0%)
The fluoroscopic puncture was selected because of the poor visibility with ultrasound	4 (18.2%)
Puncture route	
Ventral to the first rib	1 (4.5%)
First intercostal space	16 (72.7%)
Second intercostal space	5 (22.7%)
Procedure	
Technical success	22 (100%)
Procedure time (min)	
Median (range)	25 (15–40)
Complications	
Bloody sputum (CIRSE classification Grade 2)	1 (4.5%)
Drain placed (Fr)	
Median (range)	9.25 (8–20)
Drainage period (days)	
Median (range)	12 (3–20)
Pathological results from specimens	
Lung adenocarcinoma (40 × 34 × 36 mm)	1
Osteosarcoma (30 × 35 × 35 mm)	1
Clinical success	22 (100%)

## Discussion

All patients who underwent the craniocaudal approach were successful both technically and clinically. The median procedure time was 25 min; this was similar to the time reported by Takizawa et al. [4], which was within 20 min. Unlike their report, where all procedures were performed using fluoroscopic guidance, half of our procedures were performed using US guidance. In fact, poor visibility of superior sulcus lesion precluded the US-guided puncture and thus fluoroscopic puncture was used in 4 patients. Therefore, it might be preferable to prioritize fluoroscopic puncture.

Possible approaches to the superior sulcus lesions other than the craniocaudal approach include the conventional intercostal approach and transscapular approach. Although the safety and efficacy of the conventional intercostal approach are established, reaching the superior sulcus lesion is sometimes difficult because of the existence of normal lung parenchyma [6]. The transscapular approach has been reported to reach the superior sulcus lesions [7]. However, this approach requires penetration of the bone and is thus more invasive than the craniocaudal approach.

**Fig. 2:**
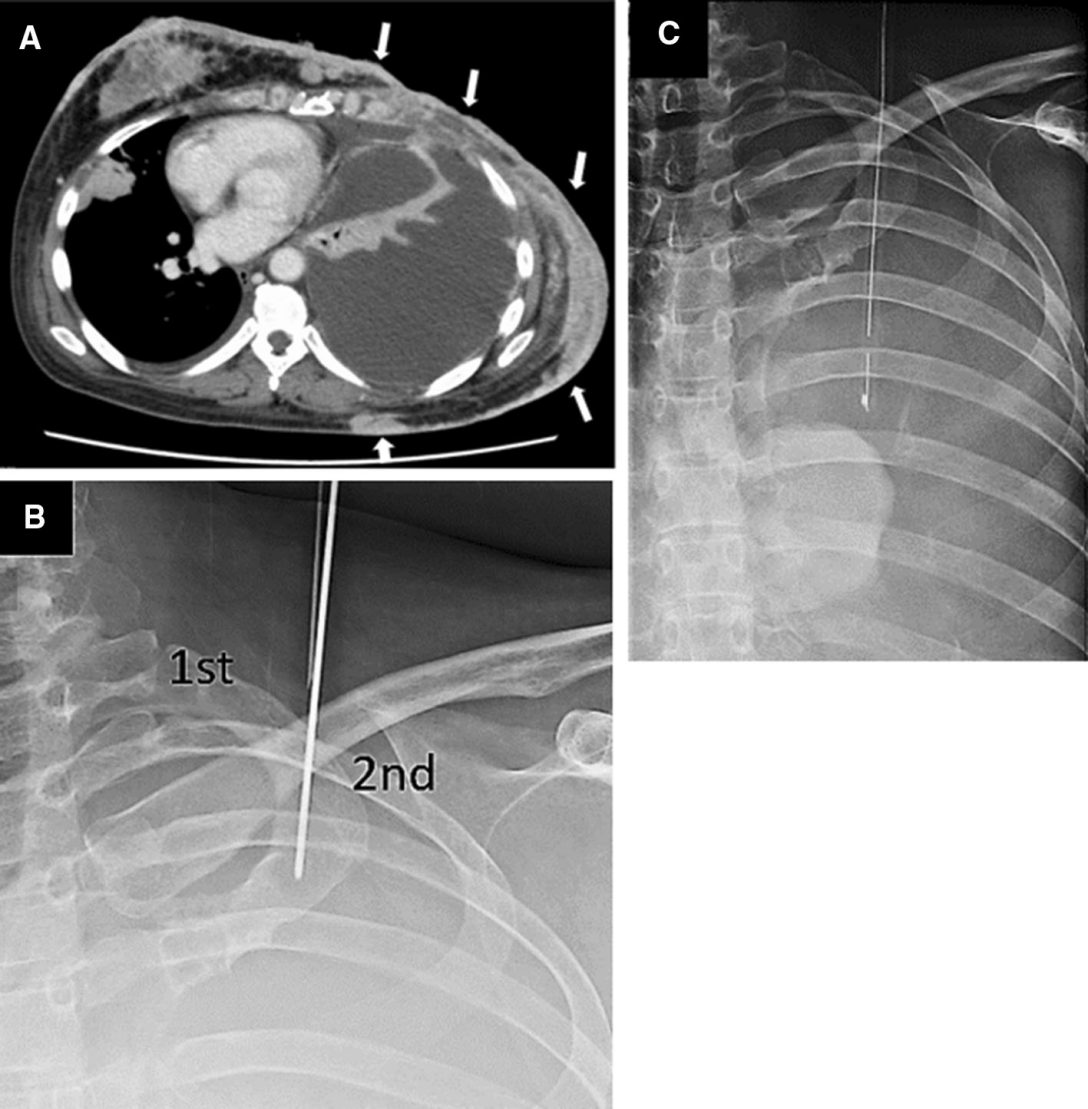
Drainage for the patient with a left pleural effusion. **A** Postoperative left breast cancer. The conventional intercostal approach is not feasible because of diffuse pleural dissemination and subcutaneous metastases (white arrow). **B, C** The needle is passed through the first intercostal space and a 16-Fr trocar catheter is placed

The craniocaudal approach has two advantages. The first one is the ability to achieve the procedure in the supine position. Therefore, this approach may be feasible for patients who are connected to ventilators, which can make changing their postures difficult. The second one is the effectiveness for the patients with diffuse subcutaneous metastases or pleural dissemination (Fig. 2). If the conventional intercostal approach is performed for such patients, it may induce needle tract seeding. Furthermore, it is extremely painful to place a drainage catheter through metastases because the local anesthesia is not very effective. Moreover, drainage tube fixation is difficult when passed through percutaneous metastases. For these reasons, it is unfavorable to puncture through the tumor.

There is no other contraindication for the craniocaudal approach than the conventional interventional radiology procedures, which includes severe thrombocytopenia or bleeding tendency.

No major complications were observed in this study, although the craniocaudal approach poses the risk of vascular injury. The needle passes through the trapezius, serratus anterior, and levator scapulae muscles, followed by the first or second intercostal space to finally reach the thoracic cavity. Therefore, injury to the small branches of the subclavian artery might occur [8].

Regarding nerve injuries, C5 and C6 of the brachial plexus are located near the needle tract. However, these structures typically run between the anterior and middle scalene muscles, which are anterior to the needle tract of the axial puncture approach [9, 10]. Thus, the possibility of injuring the brachial plexus is considered low.

This study was subject to several limitations. First, it included a limited number of patients from a single center. Second, it was retrospective study and thus might have the inherent biases. Third, inferior visibility with US might be caused by old US equipment, but it was difficult to collect enough information about the specification of US at that time.

## Conclusion

We reported on the findings from the use of a craniocaudal approach for superior sulcus lesions. For patients unsuitable for the conventional intercostal approach, the craniocaudal approach could be a feasible and safe option.

## Abbreviations

US: Ultrasound
CT: Computed tomography
CIRSE: Cardiovascular and interventional radiological society of Europe

## References

[1] Shen KR, Bribriesco A, Crabtree T (2017). The American Association for Thoracic Surgery consensus guidelines for the management of empyema. J Thorac Cardiovasc Surg.

[2] Westcott JL (1985). Percutaneous catheter drainage of pleural effusion and empyema. AJR Am J Roentgenol.

[3] Klein JS, Schultz S, Heffner JE (1995). Interventional radiology of the chest: image-guided percutaneous drainage of pleural effusions, lung abscess, and pneumothorax. AJR Am J Roentgenol.

[4] Takizawa K, Nakajima Y, Ogawa Y (2011). A new method of an axial puncture approach for draining loculated pleural effusions. Cardiovasc Intervent Radiol.

[5] Filippiadis DK, Binkert C, Pellerin O, Hoffmann RT, Krajina A, Pereira PL (2017). Cirse quality assurance document and standards for classification of complications: the cirse classification system. Cardiovasc Intervent Radiol.

[6] Mercaddi CJ, Lanes SF (2013). Ultrasound guidance decreases complications and improves the cost of care among patients undergoing thoracentesis and paracentesis. Chest.

[7] Rebonato A, Maiettini D, Andolfi M (2018). CT-guided percutaneous trans-scapular lung biopsy in the diagnosis of peripheral pulmonary lesion nodules of the superior lobes using large needles. Cardiovasc Intervent Radiol.

[8] Ikka L, Mihalea C, Ben Achour N, Abdel Khalek H, Vacher C (2016). The origin of the dorsal scapular artery: anatomic variations and surgical applications. Surg Radiol Anat.

[9] Orebaugh SL, Williams BA (2009). Brachial plexus anatomy: normal and variant. ScientificWorldJournal.

[10] Harry WG, Bennett JD, Guha SC (1997). Scalene muscles and the brachial plexus: anatomical variations and their clinical significance. Clin Anat.

